# Experimental Infection of Horses with Hendra Virus/Australia/Horse/2008/Redlands

**DOI:** 10.3201/eid1712.111162

**Published:** 2011-12

**Authors:** Glenn A. Marsh, Jessica Haining, Timothy J. Hancock, Rachel Robinson, Adam J. Foord, Jennifer A. Barr, Shane Riddell, Hans G. Heine, John R. White, Gary Crameri, Hume E. Field, Lin-Fa Wang, Deborah Middleton

**Affiliations:** Commonwealth Scientific and Industrial Research Organization Livestock Industries, Geelong, Victoria, Australia (G.A. Marsh, J. Haining, T.J. Hancock, R. Robinson, A.J. Foord, J.A. Barr, S. Riddell, H.G. Heine, J.R. White, G. Crameri, L.-F. Wang, D. Middleton);; Queensland Centre for Emerging Infectious Disease, Coopers Plains, Queensland, Australia (H.E. Field)

**Keywords:** Hendra virus, infection, horses, viruses, zoonoses, Australia, HeV, experimental

## Abstract

Hendra virus (HeV) is a highly pathogenic zoonotic paramyxovirus harbored by Australian flying foxes with sporadic spillovers directly to horses. Although the mode and critical control points of HeV spillover to horses from flying foxes, and the risk for transmission from infected horses to other horses and humans, are poorly understood, we successfully established systemic HeV disease in 3 horses exposed to Hendra virus/Australia/Horse/2008/Redlands by the oronasal route, a plausible route for natural infection. In 2 of the 3 animals, HeV RNA was detected continually in nasal swabs from as early as 2 days postexposure, indicating that systemic spread of the virus may be preceded by local viral replication in the nasal cavity or nasopharynx. Our data suggest that a critical factor for reducing HeV exposure risk to humans includes early consideration of HeV in the differential diagnosis and institution of appropriate infection control procedures.

## Abstract 2

Early consideration of HeV and institution of infection control are critical for reducing human risk.

Hendra virus (HeV) is a zoonotic paramyxovirus harbored by Australian mainland flying foxes, from which it is believed to be transmitted directly to horses. In horses, HeV causes a severe, often fatal, febrile illness associated with respiratory and neurologic signs (*1*). Since its emergence in Queensland, Australia, in 1994, HeV infection of horses has regularly recurred. Of the 32 equine outbreaks, 5 have extended to involve infection of humans; of the 7 known human case-patients, 4 have died. Human infection has typically occurred after close contact with infected horses, usually horses in the terminal stages of disease or at postmortem examination, except for 1 person for whom epidemiologic findings suggested the most likely exposure to an infected horse occurred during incubation (*2*). Currently, HeV is an unmanaged emerging infectious disease.

Since the serious zoonotic potential of HeV was confirmed, clinical and laboratory evaluation of disease horses from outbreaks has been limited. In particular, the relationship between the onset of clinical signs and duration of viral shedding has not been determined, and the understandably few equine experimental infection studies conducted in the mid-1990s (*3*) yielded limited data that could guide effective management of the exposure risk to humans.

Further concern arose after an HeV outbreak in the Brisbane suburb of Thornlands (Redlands Shire), Queensland, in 2008, in which the major clinical signs in horses were attributable to disease of the central nervous system (*4*). Although nervous system signs have been associated with previous outbreaks, HeV is more commonly considered to induce a respiratory syndrome in horses. In the Redlands 2008 outbreak, credible alternate provisional diagnoses and thus delay in definitive diagnosis likely contributed to an increased HeV exposure risk to attending staff and to in-contact horses; 2 staff members became infected, 1 fatally (*4*).

The objectives of this study were to monitor potential routes of shedding for evidence of HeV replication in horses experimentally exposed to Hendra virus/Australia/Horse/2008/Redlands and to compare the associated clinical syndrome with that observed after infection with the HeV isolate from the first outbreak in 1994. These data would provide a framework for assessing the relative transmission risk posed by horses at various times during acute HeV infection and permit incorporation of recommendations for reducing the transmission risk to humans and other horses into advisory and outbreak management strategies. Following the observation in the Redlands outbreak of a predominantly neurologic disease, an experimental challenge was carried out under BioSafety Level 4 conditions at the Australian Animal Health Laboratory, Geelong, Victoria, Australia, in late 2008.

## Methods

### Animals

Three mares were housed in individual pens within 1 room at BioSafety Level 4. Room temperature was maintained at 22°C with 15 air changes per hour; humidity varied from 40% to 60%. Animal husbandry methods and experimental design were endorsed by the Commonwealth Scientific and Industrial Research Organization, Australian Animal Health Laboratory and Animal Ethics Committee, and aligned with the Code of Practice for the Welfare of Horses (Bureau of Animal Welfare, Victoria, Australia).

Horses were acclimated to the facility for 1 week before exposure to HeV. Immediately before viral challenge, each mare was fitted with an intrauterine (transcervical) temperature transponder to continuously record core body temperature (*5*). An indwelling catheter was placed in the jugular vein of each animal and sutured in position*.*

Horses were clinically assessed at least 2×/d, and when a predetermined humane endpoint was reached, they were euthanized. The humane endpoint was defined as fever accompanied by depression or other signs consistent with localization to respiratory or neurologic systems. This clinical assessment permitted documentation of the incubation period, duration and route of virus shedding from the time of virus exposure to readily detectable disease, and time for localization of virus to various body tissues.

### Virus

HeV (Hendra virus/Australia/Horse/2008/Redlands) was isolated on Vero cells from the spleen from a horse with naturally acquired disease in the Redlands outbreak. Genetic analysis of this isolate showed 99.6% similarity at the amino acid level to the original HeV isolate (Hendra virus/Australia/Horse/1994/Hendra) (*6*). Because the median infectious dose of HeV in horses is not known, the virus challenge dose was selected to mimic previous experimental studies (*3*); virus (2 × 10^6^ 50% tissue culture infectious dose) was administered oronasally to each horse.

### Clinical Data and Biological Sample Collection and Analysis

Clinical observations were recorded 2×/d beginning 3 days before HeV exposure and included measurement of rectal temperature (to augment records from the implanted transponders) and heart rate measured with an electronic monitor. The character of the respiratory pattern and effort was also noted on each occasion (resting respiratory rate had previously been assessed as highly variable from time to time in each animal and so was not routinely recorded).

Biological samples (nasal, oral, and rectal swab specimens and blood) were collected every day. Urine and feces were collected from the pen floor and mid-stream urine was collected on an opportunistic basis. All biological samples, except blood for serologic tests, were placed immediately on wet ice and then stored at –80°C until processed. Serum samples were stored at –20°C until tested for antibody against HeV.

Nasal, oral, and rectal swab specimens; urine; feces; and blood were analyzed for HeV nucleic acid by quantitative real-time PCR directed against the N and P genes (*7*) and for live HeV by virus isolation in Vero cells. After being euthanized, each horse underwent postmortem examination. Diverse samples were retained for virus isolation, real-time PCR, histopathologic examination, and immunohistochemical testing by using rabbit α-Hendra N-protein antiserum.

## Results

### Clinical Observations

#### Horse 1

**Figure 1:**
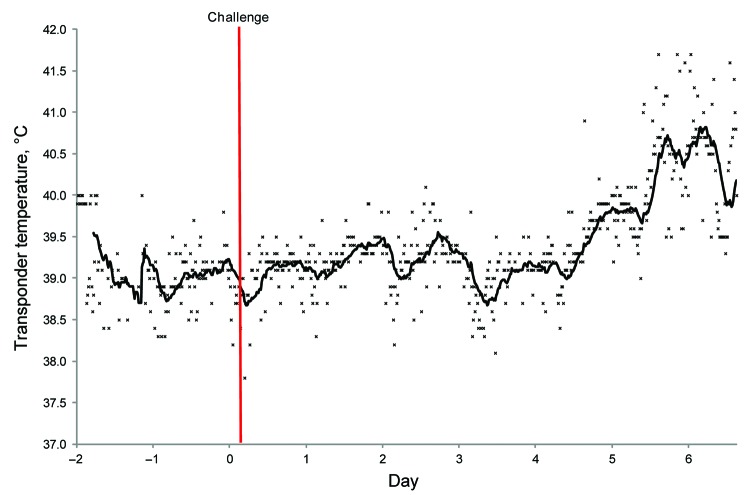
Temperature transponder data for horse 1 during experimental infection with Hendra virus, Australia. Before viral challenge, each mare was fitted with an intrauterine (transcervical) temperature transponder to allow continuous recording of core body temperature. Temperature was measured every 15 minutes in each horse. Solid line represents the moving average based on 20 temperature readings.

**Figure 2:**
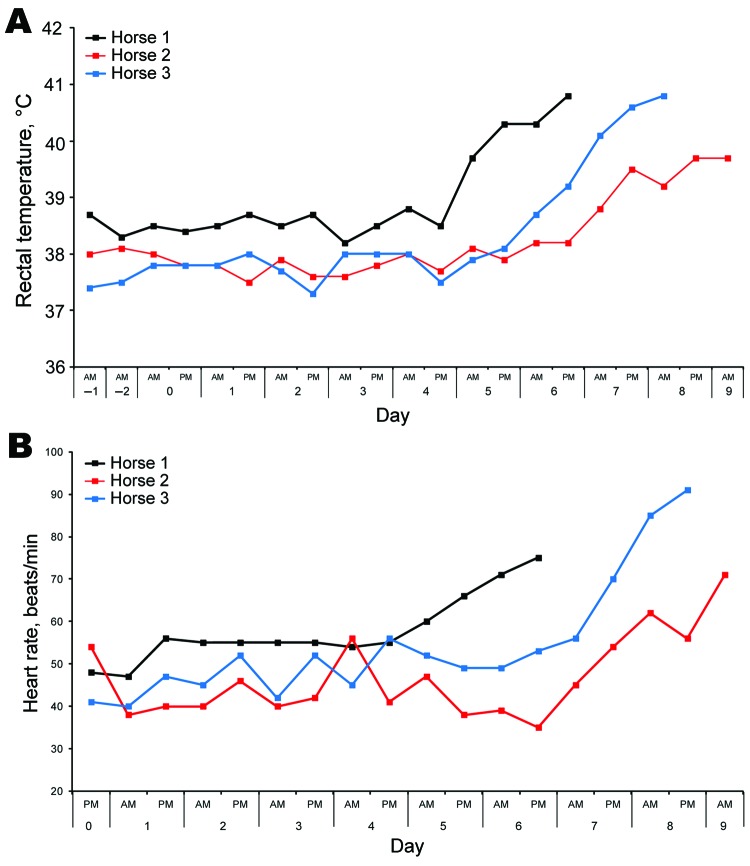
Rectal temperatures (A) and heart rates (B) of each horse after experimental infection with Hendra virus, Australia. Data were collected by using an electronic monitor 2×/d, along with comments on general demeanor. Data were used to determine a humane endpoint for each animal.

The horse remained well until day 5 postchallenge, when its temperature began to rise (Figure 1) in parallel with a steady rise in resting heart rate (Figure 2). At this time the horse was eating well and otherwise appeared clinically normal.

On day 6, the horse was clinically depressed with reduced appetite. Temperature and heart rate had continued to rise, and over several hours, the horse exhibited continuous restlessness, with constant shifting of weight between all 4 limbs, especially the hind limbs. By afternoon, the horse was disinterested in its surroundings and had begun to stand with its head facing the side of the pen. The horse was euthanized, and postmortem examination was conducted.

#### Horse 2

A slight bilateral serous nasal discharge was observed in this horse on day 2 postchallenge. The horse remained otherwise well until day 7, when a temperature above baseline developed in parallel with a rise in resting heart rate (Figure 2). At this time, the horse did not exhibit any other abnormal clinical signs. The following day, the horse was slightly depressed, and the elevated heart rate and temperature appeared to have stabilized. However, on day 9, the heart rate continued to rise, and the horse exhibited mild dyspnea with a prolonged expiratory phase. This normally quiet mare also became agitated when approached. The horse was euthanized on the afternoon of day 9, and postmortem examination was conducted.

#### Horse 3

A slight bilateral serous nasal discharge was observed in this horse on day 2 postchallenge. The horse remained otherwise well until day 6 when its temperature began to rise above baseline. Fever was established by day 7, and a concomitant rise in heart rate was also noted (Figure 2). The serous nasal discharged had resumed, but the horse was otherwise well and eating normally. On day 8, temperature and heart rate were continuing to rise, and small amounts of blood coated in mucus were seen in the feces. The mare exhibited a rigid forelimb stance, alternating with general restlessness and constant shifting of weight from limb to limb, difficulty eating, frequent head shaking, and irritability with attempts to bite her handlers. A panting type of respiration was noted. The horse was euthanized on the afternoon of day 8.

### Postmortem and Histopathologic Findings

#### Horse 1

Significant gross abnormalities comprised enlarged and edematous submandibular lymph nodes, and several 2 × 0.5–cm subpleural hemorrhages were noted on the left lung. A small (6 × 6 cm) area of brownish pink consolidation was present on the ventral border of the left lung posterior to the cardiac notch. A 2.5-cm follicle was noted in the left ovary.

On histologic examination, systemic vasculitis was observed that affected meninges, nasal mucosa, trachea, lung, diverse lymph nodes, spleen, kidney, heart, uterus, ovary, and intestine. Edema, syncytial cells, viral inclusion bodies, and alveolitis were seen in lung sections. Focal necrosis of the adrenal gland was identified, together with glomerulitis and syncytial cell formation in the kidney. HeV antigen was detected in tissues and organs, including meninges, alveolar walls, lymph nodes, renal glomeruli, and adrenal glands and in blood vessels supplying each of these. In addition, the nasal mucosa, trachea, spleen, heart, uterus, ovary, and intestine showed HeV antigen.

#### Horse 2

Significant gross abnormalities comprised enlarged and edematous submandibular and bronchial lymph nodes and heavy lungs that oozed fluid from the cut surface. Numerous petechial hemorrhages were found over the surface of the diaphragmatic regions of the lung. The liver was small with an irregular finely nodular surface.

**Figure 3:**
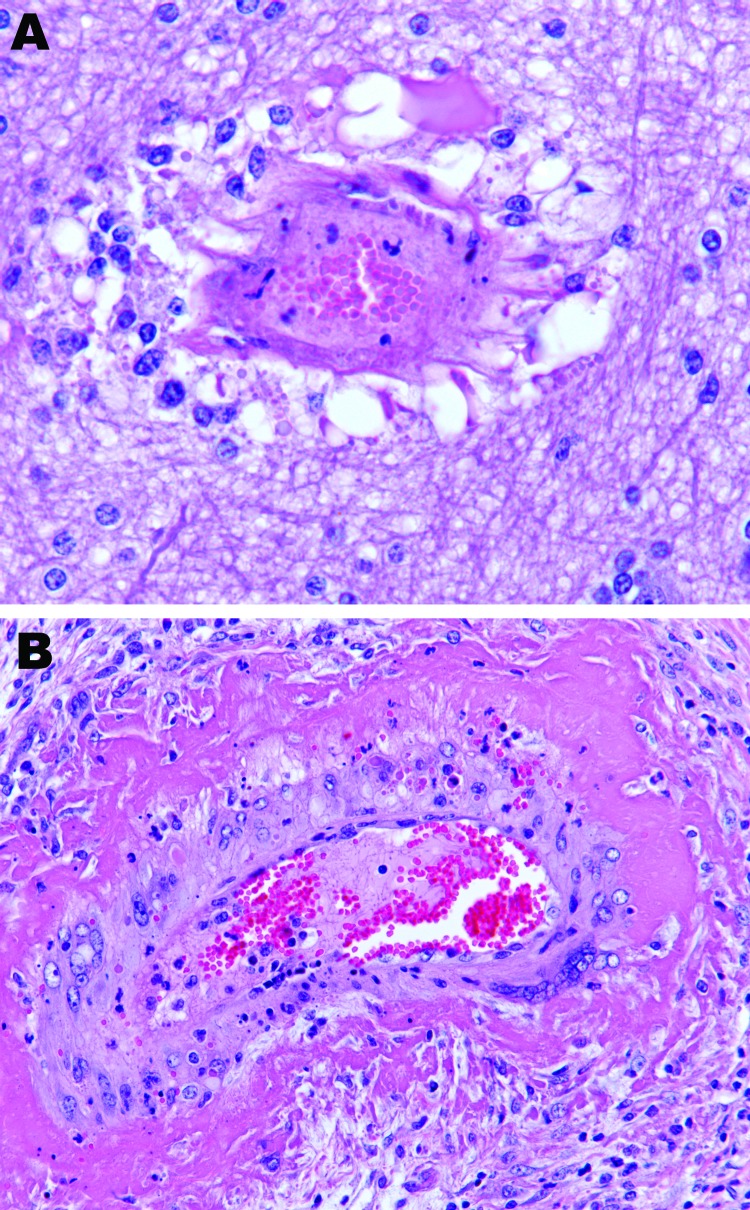
Brain vasculitis in horse experimentally infected with Hendra virus, Australia. A) Parenchyma and B) ovary of horse 2. Original magnification ×200.

**Figure 4:**
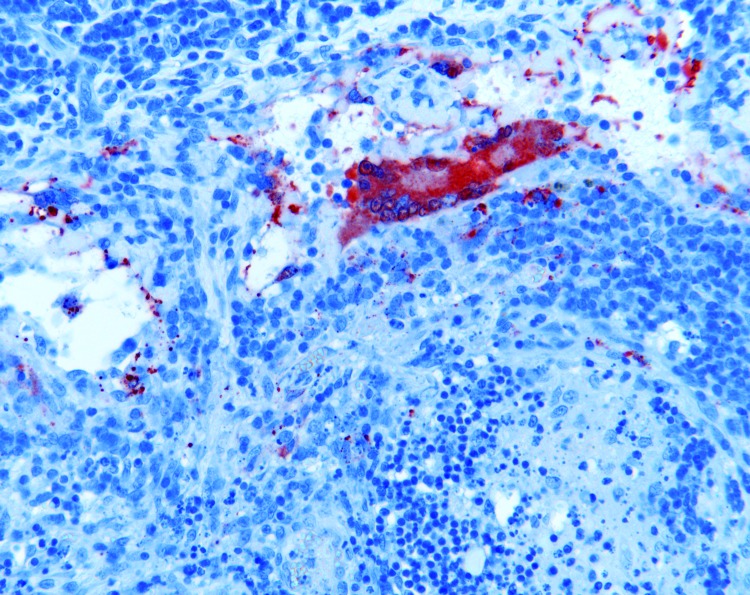
Lymphadenitis with syncytial cell formation in horse 2 experimentally infected with Hendra virus (HeV), Australia. Immunohistochemical staining of HeV N protein showing presence of antigen in red. Original magnification ×200.

On histologic examination, systemic vasculitis was observed that affected meninges, brain (Figure 3, panel A), nasal mucosa, trachea, lung, diverse lymph nodes, spleen, liver, kidney, heart, uterus, ovary (Figure 3, panel B), and intestine. Edema, syncytial cells, viral inclusion bodies, and alveolitis were seen in lung sections. Focal necrosis and syncytial formation within lymph nodes were identified, together with glomerulitis and syncytial cell formation in the kidney. Acute myocarditis and focal necrosis of corpus luteum tissue were also identified. HeV antigen was detected in tissues and organs, including meninges, alveolar walls, lymph nodes (Figure 4), renal glomeruli, myocardium, and ovary and in blood vessels supplying each of these. Again, HeV antigen was detected in nasal mucosa, liver, spleen, adrenal gland, uterus, and intestine. Hepatic amyloidosis was also noted but was considered to be an incidental finding.

#### Horse 3

**Figure 5:**
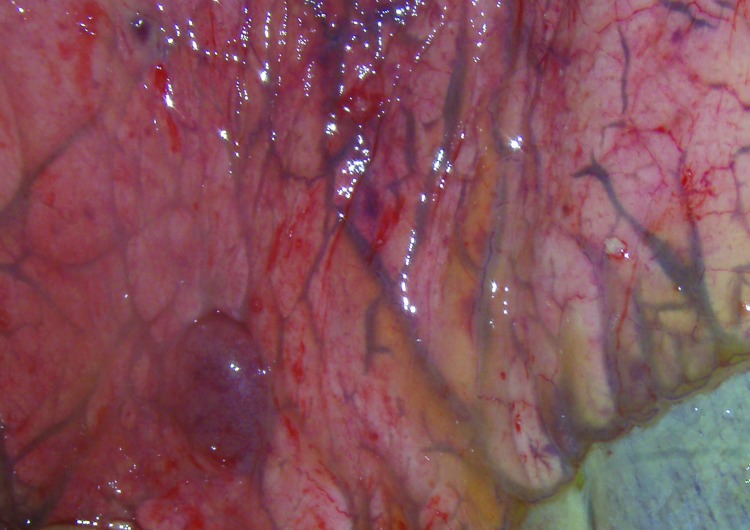
Dilation of lymphatic vessels and ventral lung lobe margins of horse 3 experimentally infected with Hendra virus, Australia. Original magnification ×10.

At postmortem examination, we identified swollen and edematous submandibular, sternal, and bronchial lymph nodes and dilation of lymphatic vessels at ventral lung lobe margins (Figure 5). We also found endometrial edema with purplish discoloration of the serosal surface of the uterus.

On histologic examination, systemic vasculitis was observed affecting meninges, nasal mucosa, lung, diverse lymph nodes, tonsil, spleen, liver, kidney, heart, uterus, ovary, and intestine. Edema, syncytial cells, viral inclusion bodies, and alveolitis were seen in lung sections. Focal necrosis and syncytial formation within lymph nodes was identified, together with glomerulitis and syncytial cell formation in the kidney. Acute myocarditis and focal necrosis of adrenal and corpus luteum tissue was detected. HeV antigen was also detected in tissues and organs including alveolar walls, lymph nodes, tonsil, renal glomeruli, myocardium, and ovary and in blood vessels supplying each of these, as well as in nasal mucosa, liver, spleen, adrenal gland, uterus, and intestine.

### Virus Loads in Clinical Samples

Viral genetic material was detected in nasal swabs from 2 days postchallenge (Table, only P gene data shown), consistently in 2 of the animals and intermittently in the third. The steady increase in relative copy numbers over time is consistent with viral replication in the upper respiratory tract and shedding into the nasal cavity in nasal secretions. Viral RNA was first found in the blood of each horse at least 1 day before onset of fever. After onset of fever, but before development of other clinical signs of illness, HeV genome was detected in the oral swabs, urine, and feces of each horse; the rectal swab only of horse 2 was positive. Fecal material on the floor of the pen could have been contaminated by urine containing viral genetic material. In addition, the smaller amount of material collected on the rectal swab could have influenced sensitivity of the test. Once clinical disease was established, all samples had detectable levels of HeV genome, except the rectal swabs of horses 1 and 3. All samples in which viral RNA was found were examined for live virus by passage in Vero cells. Virus was not reisolated from any sample collected before postmortem examination. Blood samples collected during acute disease, as well as samples of urine and feces, were highly toxic to tissue cultures, and virus might have been present at low titer in some of these samples.

**Table:** Real-time PCR detection of HeV in daily shedding samples in experimental infection of horses, Australia, 2008*

Animal/sample	Cycle threshold values from HeV P gene real-time RT-PCR†									
	Days postinfection									
	0	1	2	3	4	5	6	7	8	9
Horse 1										
Blood	−	−	−	−	39.4	33.0	31.2			
Urine	−	−	−	41.9	−	41.6	36.2			
Feces	−	−	−	−	−	40.7	36.1			
Rectal swab	−	−	−	−	−	−	42.0			
Nasal swab	−	−	37.5	34.7	35.9	29.5	32.8			
Oral swab	−	−	−	−	−	41.2	38.5			
Horse 2										
Blood	−	−	−	−	−	−	37.3	32.2	31.4	29.9
Urine	−	−	−	−	−	−	−	36.2	36.3	33.5
Feces	−	−	−	−	−	−	40.7	37.0	35.0	35.2
Rectal swab	−	−	−	−	−	−	−	43.6	36.4	36.5
Nasal swab	−	−	36.3	32.4	38.9	34.3	31.1	28.1	29.2	35.2
Oral swab	−	−	−	−	−	−	−	36.6	35.8	34.5
Horse 3										
Blood	−	−	−	−	−	39.1	36.3	32.8	31.6	
Urine	−	−	−	−	−	−	40.7	39.0	34.3	
Feces	−	−	−	−	42.2	−	39.2	35.3	34.5	
Rectal swab	−	−	−	−	−	−	−	42.9	42.8	
Nasal swab	−	−	42.0	NA	41.4	43.0	−	37.9	32.9	
Oral swab	−	−	−	−	41.4	41.8	40.3	39.5	38.5	

*HeV, Hendra virus; RT-PCR, reverse transcription PCR; −, negative by real-time PCR testing; NA, sample not available for testing. Shading indicates that horse was euthanized. †Individual samples were taken, RNA extracted, and samples tested by real-time PCR in duplicate.

### Virus Loads in Tissue Samples

**Figure 6:**
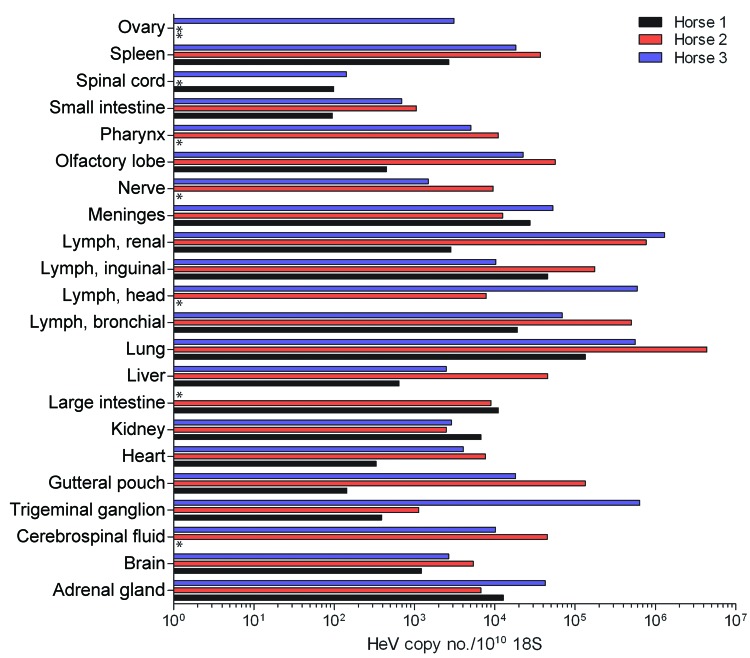
Relative abundance of Hendra virus (HeV) P RNA in different horse tissues at postmortem examination after experimental infection with HeV, Australia. Values are expressed relative to ribosomal 18S copies. Tissue origins are indicated along the y-axis. *Sample not available for testing.

Viral RNA (N and P genes) was detected in all tissues sampled at postmortem examination (Figure 6, only P gene data displayed). Reisolation of virus was attempted for all tissues; tissues from which virus was recovered generally were those with the highest levels of target genes. From horse 1, these tissues were kidney; lung; and submandibular, inguinal, and renal lymph nodes. From horse 2, virus was recovered from the guttural pouch; pharynx; submandibular, inguinal, bronchial, and renal lymph nodes; lung; spleen; kidney; heart; large intestine; spinal cord; brain; and intrathoracic sympathetic chain. From horse 3, virus was recovered from the guttural pouch; submandibular, inguinal, bronchial, and renal lymph nodes; lung; kidney; heart; adrenal gland; spinal cord; brain; cerebrospinal fluid; and meninges.

## Discussion

The mode and critical control points of HeV spillover from flying foxes to horses, along with the risk for transmission of virus from infected horses to other horses and to humans, is poorly understood. In this study, we successfully established systemic HeV in 3 horses exposed to 2 × 10^6^ 50% tissue culture infectious dose HeV (Hendra virus/Australia/Horse/2008/Redlands) oronasally. In 2 of the 3 animals, HeV RNA was continually detected in nasal swabs over the course of the incubation period, strongly suggesting that systemic spread of virus may be preceded by local viral replication in the nasal cavity or nasopharynx.

These data indicate that nasal secretions of asymptomatic horses may pose a transmission risk during the early phase of disease that precedes viremia, fever, or other discernable clinical signs of HeV infection. However, the increasing gene copy number recovered over time also suggests that the risk provided by these animals is relatively low, compared with animals in the immediate presymptomatic and symptomatic stages of infection. Duration of exposure also contributes to infection risk because longer contact time increases the potential for acquisition of an infectious dose of virus. Additionally, certain types of contact or procedures may contribute to infection, such as nasal intubation or routine dental procedures, where operator risk is increased even in the preclinical stage of infection.

The febrile, and then symptomatic, horse likely poses a greater transmission risk not only from virus shed in its nasal secretions but also from excretions, such as urine, and blood. However, the activity likely to pose the highest transmission risk is postmortem examination of a horse that has died of acute HeV infection. The potentially high virus load in the animal at this time provides a scenario for gross contamination of operator and assistants with infective material and the associated additional risk inherent in the handling of sharp instruments. Of the 7 known human HeV infections, 2 have been associated with postmortem examination of affected horses (*8**,**9*) and the remainder with contact with clinically ill horses in the late incubation period (*2**,**10*).

Clinical signs observed in our study, including fever, tachycardia, inappetence, depression, dyspnea, and restlessness, were generally consistent with signs recorded in the first HeV outbreak (*10**–**12*) as well as earlier experimental studies that used the original HEV isolate (Hendra virus/Australia/Horse/1994/Hendra) (*13*). The early field observations also mention ataxia (*10**,**14*) and myoclonus (*14*), which suggest that neurologic presentations are regularly associated with HeV disease in horses. Similarly, nonsuppurative meningoencephalitis is commonly found in experimentally induced infection (*15*), and the florid neurologic signs noted in field cases in the Redlands 2008 outbreak (*4*) might merely reflect the normal spectrum of HeV in horses. Although the route of natural infection of horses by HeV is not known, primary exposure likely occurs through the upper respiratory tract or the oropharynx. In previous experimental studies using HeV, either route has been used to successfully establish infection and therefore was selected for the current study. Notably, 3 of 5 horses at Redlands had medical problems involving the head (corneal lesion, nasal granuloma, and mandibular fracture) (*4*), which created the potential for an exposure route that bypassed mucosal protective mechanisms. If so, these routes of infection may also have influenced the course of infection.

Our data suggest that a critical part of reducing HeV exposure risk to veterinarians and animal attendants includes early consideration of HeV in the differential diagnosis of the febrile horse, with institution of appropriate infection control procedures, at least until a definitive diagnosis is obtained. Accordingly, published guidelines on handling potential HeV cases have been revised to account for this improved understanding of the dynamics of HeV infection in the horse (*1*). Most febrile horses are not infected with HeV but will often require ongoing veterinary interventions for underlying disease. We acknowledge that case management under these circumstances is not straightforward, particularly with respect to suitable personal protective equipment, because neither the human infectious dose nor the virus load in the air are known.

In spite of attendant risk, postmortem examination of affected animals remains particularly valuable when atypical disease is observed or in other situations where a diagnosis is essential, such as when human exposure is suspected or transmission to other animals might have occurred. Necropsy can be conducted safely by suitably experienced and equipped operators with predetermined strategies and infrastructure for personnel and environmental decontamination. A level of risk reduction should be adopted for inexperienced operators or those ill prepared to safely manage such a procedure, especially where diagnostic confirmation of a typical HeV case is sought. In such cases limited collection of tissues, such as superficial lymph nodes, may offer a tolerable balance between the value of diagnostic confirmation and the infection risk associated with achieving it.
